# Spontaneous Necrosis of a High-Risk Bladder Tumor Under Immunotherapy for Concurrent Malignant Melanoma: Role of BRAF Mutations and PD-L1 Expression

**DOI:** 10.3390/biomedicines13020377

**Published:** 2025-02-05

**Authors:** Cristian Condoiu, Mihael Musta, Alin Adrian Cumpanas, Razvan Bardan, Vlad Dema, Flavia Zara, Cristian Silviu Suciu, Cristina-Stefania Dumitru, Andreea Ciucurita, Raluca Dumache, Hossam Ismail, Dorin Novacescu

**Affiliations:** 1 Doctoral School, Victor Babes University of Medicine and Pharmacy Timisoara, E. Murgu Square, No. 2, 300041 Timisoara, Romania; cristian.condoiu@umft.ro (C.C.); vlad.dema@umft.ro (V.D.); andreea.ciucurita@umft.ro (A.C.); 2 Department XV, Discipline of Urology, Victor Babes University of Medicine and Pharmacy Timisoara, E. Murgu Square, No. 2, 300041 Timisoara, Romania; cumpanas.alin@umft.ro (A.A.C.); razvan.bardan@umft.ro (R.B.); 3 Department II of Microscopic Morphology, Victor Babes University of Medicine and Pharmacy Timisoara, E. Murgu Square, No. 2, 300041 Timisoara, Romania; flavia.zara@umft.ro (F.Z.); cristi.suciu@umft.ro (C.S.S.); cristina-stefania.dumitru@umft.ro (C.-S.D.); novacescu.dorin@umft.ro (D.N.); 4 Department VIII, Discipline of Forensic Medicine, Bioethics, Deontology and Medical Law, Victor Babes University of Medicine and Pharmacy Timisoara, E. Murgu Square, Nr. 2, 300041 Timisoara, Romania; raluca.dumache@umft.ro; 5 Center for Ethics in Human Genetic Identifications, Victor Babes University of Medicine and Pharmacy Timisoara, E. Murgu Square, No. 2, 300041 Timisoara, Romania; 6 Department of Urology, Lausitz Seenland Teaching Hospital, University of Dresden, Maria-Grollmuß-Straße, No. 10, 02977 Hoyerswerda, Germany; drismailhossam@gmail.com

**Keywords:** urothelial carcinoma, bladder cancer carcinogenesis, immunotherapy, targeted therapies, multiple concomitant neoplasms, treatment response prediction biomarkers, programmed death ligand 1 (PD-L1) combined positive score, *BRAF* gene mutations, BRAF V600E positive malignant melanoma

## Abstract

**Background:** Bladder cancer (BC) is a heterogeneous malignancy, and predicting response to immune checkpoint inhibitors (ICIs) remains a challenge. Herein, we investigate a high-risk bladder tumor, which developed during anti-BRAF/MEK therapy for a concurrent advanced *BRAF-V600E*-positive malignant melanoma (MM) and subsequently underwent complete spontaneous necrosis following Nivolumab immunotherapy, only to recur thereafter while still under the same treatment. This unique scenario provided an opportunity to investigate the roles of *BRAF* gene mutations in BC pathogenesis, respectively, of PD-L1 expression in immunotherapy response prediction. **Methods:** We retrospectively analyzed BC specimens obtained via transurethral resection at two critical time-points: prior to the complete spontaneous necrosis under Nivolumab (prenecrosis) and after tumor recurrence postnecrosis (postnecrosis). The *BRAF* gene mutation status was evaluated using quantitative polymerase chain reaction (qPCR). PD-L1 expression was assessed by immunohistochemistry (IHC), quantified using the combined positive score (CPS), and a cutoff of ≥10 for positivity. **Results:** Neither pre- nor postnecrosis BC samples harbored *BRAF* gene mutations. Prenecrosis PD-L1 expression (CPS = 5) indicated a minimal likelihood of response to immunotherapy. However, complete spontaneous necrosis occurred under Nivolumab, followed by recurrence with further reduced PD-L1 expression (CPS = 1). **Conclusions:** The complete BC regression challenges the conventional role of PD-L1 as a sole predictive biomarker for immunotherapy. This study also highlights the potential role of BRAF/MEK inhibitors in BC oncogenesis and underscores the need for alternative biomarkers, such as tumor mutation burden (TMB) and circulating tumor DNA (ctDNA), to guide treatment selection in BC better.

## 1. Introduction

Bladder cancer (BC) is the 10th most common cancer globally and one of the more prevalent genitourinary malignancies, with the second highest mortality rate after kidney cancer and a steadily increasing incidence, especially in developed countries. With 550,000 newly diagnosed cases and nearly 200,000 lives claimed in 2018 alone, BC remains a significant and rising contributor to the global cancer burden, despite survival rates having been improved with earlier diagnosis, robotic surgical techniques, and the introduction of immunotherapy [1].

In intimate contact with the stored urine, the apical region of the urothelium is comprised of a single row of specialized “umbrella” cells, frequently binucleated, with tight intercellular junctions and a proteic covering (uroplakines), forming an impermeable, mechanical, and osmotic barrier between the bladder content and underlying tissues [2]. The urothelial cells constituting the bladder mucosa are thus constantly exposed to potentially mutagenic environmental agents, which are being filtered into the urine by the kidneys [3]. Unsurprisingly, ~90% of BC cases, especially in the developed world, arise from these urothelial cells, i.e., urothelial carcinoma. Moreover, despite occurring mostly in the bladder, urothelial carcinoma may also be present, albeit on rare occasions, elsewhere in the urinary tract, i.e., proximal urethra, ureters, and pyelocaliceal systems [4].

In fact, BC represents the culmination of a continuous spectrum of tissue transformations, typically initiated by external aggressive factors. Genomic injuries alter intracellular metabolic signaling pathways, leading to the emergence of mutant cell clones with superior reproductive capabilities unhindered by cytotoxic immune monitoring, as opposed to surrounding normal cells. This proliferation will ultimately manifest clinically, yet the transition from normal epithelium to neoplasia occurs gradually. The mutant cell clone initially proliferates and causes hyperplasia, i.e., increased cellularity without pathological nuclear changes. Subsequently, as the urothelial lesion further progresses and consolidates into neoplasia, nuclear atypia becomes apparent [5]. The degree of nuclear atypia is quantified in clinical practice, constituting an essential component of pathological staging known as the G parameter [6].

Beyond occupational risk factors such as exposure to aromatic amines and lifestyle-related factors like smoking, certain therapeutic interventions have been implicated in BC etiopathogenesis. The oncogenic effect of ionizing radiation on the urothelium has been conclusively demonstrated in atomic bomb survivors and shows clear dose dependence, with exposures >1 Gy being associated with a 60% increase in risk [7]. Postradiation exposure BC is not age-dependent but typically appears after a significant latency period (over a decade). External beam radiotherapy for prostate adenocarcinoma increases BC risk 1.5–2-fold, while brachytherapy carries a lower risk, whereas intensity-modulated regimens associate the lowest risk profiles [8,9]. In terms of chemotherapy-induced BC, cyclophosphamide stands out as the only chemotherapeutic agent with a proven causative effect. Its primary oncogenic metabolite, phosphoramide mustard, demonstrates clear dose-dependent relationships with BC occurrence. Studies have shown a 4.5-fold increase in risk at cumulative doses of ~20 g, rising to 7-fold at doses exceeding 50 g. Here also, a latency period will be observed, as the risk increases significantly after 5 years posttreatment, reaching a peak incidence of 15% at 10–14 years post exposure [10].

Regarding carcinogenesis, urothelial carcinomas follow two distinct but somewhat overlapping pathways of tumor proliferation—papillary and nonpapillary. The papillary pathway encompasses approximately 80% of BCs, presenting as superficial exophytic papillary lesions originating from urothelial hyperplasia [11]. These tumors are typically low-grade papillary lesions that may exhibit recurrence but rarely further invade the bladder wall or metastasize [11]. On the other hand, the nonpapillary pathway represents 15–20% of tumors, featuring high-grade solid nonpapillary BCs emerging from high-grade intraurothelial neoplasia. These tumors display aggressive invasion of the bladder wall and a heightened propensity for distant metastasis [11]. Papillary tumors confined to the mucosa are classified as Ta, according to the tumor–node–metastasis (TNM) classification system [12]. Flat, high-grade tumors confined to the mucosa are classified as carcinoma in situ—CIS (Tis) [12].

The essential clinical and therapeutic distinction in bladder tumors relates to local extension within the bladder wall. Two main categories are described: non-muscle-invasive BC (NMIBC)—CIS, Ta, T1-, and muscle-invasive BC (MIBC)—T2–T4. Treatment strategies differ significantly between these groups. Non-muscle-invasive forms typically respond well to endoscopic resection, potentially followed by intravesical chemotherapy or local immunotherapy through BCG instillations. However, for muscle-invasive forms and high-risk, noninvasive forms (including pT1G3, presence of CIS, multiple tumors exceeding 3 cm, and BCG-recurrent cases), radical cystectomy represents the therapeutic gold standard [12]. Approximately 75% of BC patients present with localized disease (mucosal–Ta, CIS/submucosal–T1) at diagnosis. For patients under 40 years of age, this percentage is even higher. Overall, there is an increased prevalence of localized forms due to their lower mortality risk compared to T2–4 stages [13].

Importantly, BC demonstrates remarkable genetic heterogeneity, exhibiting the highest degree of genetic variation among urogenital neoplasms. Multiple specific mutations have been described in the literature for both recurrent non-muscle-invasive forms (pTa/pT1) and distinct urothelial origins. Beyond specific mutations, multiple intracellular signaling pathways have been implicated in tumor metabolism, contributing to its complex molecular landscape [14].

In carcinogenesis, the mitogen-activated protein kinase (MAPK) pathway stands out as a significant signaling cascade regulating cellular processes. Dysregulation of this pathway, often attributed to mutations in various mediators, is a common feature in many cancer types [15]. Herein, the *BRAF* gene, a proto-oncogene described in 2002, encodes a phosphorylating enzyme (serine/threonine-protein kinase B-Raf) involved in epidermal growth factor (EGF)-dependent intracellular signaling for initiating cell proliferation. Mutation of this gene will cause uncontrolled cell proliferation, independent of EGFs. Furthermore, the mitogen-activated protein kinase kinase (MEK) represents an effector for B-Raf and an integration point for multiple biochemical signals involved in cell proliferation and differentiation and transcription modulation [16].

The *BRAF* gene, known for somatic activating mutations, has been identified in melanomas (>60%) and other cancer types, with the V600E substitution in exon 15 being predominant [17]. The most frequently reported mutation, observed in 80% of studied malignant melanomas (MMs), involves a thymine to adenine (T→A) transversion at nucleotide 1799 (V600E), formerly denoted as T1796A (V599E) [17]. However, the role of these *BRAF* gene mutations in BC oncogenesis remains unclear.

Interestingly, *BRAF* proto-oncogene mutations represent the main oncogenetic mechanism in canine urothelial carcinoma models [18] and have long been implemented as a veterinary biomarker for BC [19,20,21]. Moreover, recent empirical evidence, albeit scarce, has seemingly implicated *BRAF* gene mutations in the carcinogenesis of urothelial carcinomas in humans as well. Even so, these are isolated multigenic mutations in the BRAF/MEK signaling pathway, which have not yet been corroborated by larger genomic initiatives, such as The Cancer Genome Atlas (TCGA) Research Network. In the current work, we aim to identify the potential oncogenic influence of BRAF/MEK inhibitors in BC from a unique clinical perspective.

Immune checkpoint inhibitor (ICI) therapy has emerged as a promising treatment modality for BC specifically, as it has found multiple clinical applications in both metastatic contexts, as well as, more recently, the neoadjuvant setting. The therapeutic principle involves activating the immune system against tumor cells by abolishing pathological interactions between tumor cells and CD8+ cytotoxic T cells. This process primarily targets two major signaling pathways, allowing for tumor cell immune evasion, namely: (1) programmed cell death protein 1 (PD1)-programmed death-ligand 1 (PD-L1); and (2) cytotoxic T-lymphocyte associated protein 4 (CTLA4)-B7 [22].

Modern therapeutic approaches utilize PD1 inhibitors (such as Nivolumab and Pembrolizumab), as well as PD-L1 inhibitors (like Atezolizumab), to interrupt this pathological signaling [22,23]. Conversely, for the CTLA4-B7 mechanism, which operates similarly but also manifests under physiological conditions, inhibitors such as Ipilimumab specifically target pathway overexpression [22,23]. Currently, due to the high costs associated with immunological treatments, patient selection for such therapies is guided by therapy response predictors, of which PD-L1 expression levels in tumor cells and inflammatory infiltrate, with a combined expression threshold of >10% typically required for initiating Nivolumab [24,25,26]. However, emerging evidence has begun to challenge this approach, suggesting the need for more nuanced patient selection criteria.

Our current paper is centered around a very unique and intriguing clinical case of a high-risk bladder tumor, occurring and progressing after the initiation of anti-BRAF/MEK chemotherapy for a concurrent, advanced, BRAF V600E positive, malignant melanoma (MM), which then demonstrated complete spontaneous bladder tumor necrosis, as a consequence of the newly instated Nivolumab immunotherapy, aimed at addressing the MM progression under chemotherapy. This clinical scenario, due to its complexity and particularities, offered multiple valuable research avenues regarding the molecular oncogenesis of BC (i.e., the apparent atypical involvement of the BRAF pathway), as well as the prediction of therapeutic response to immunotherapy. Thus, due to the unusual evolution of the BC, *BRAF* gene mutations and PD-L1 expression were retrospectively evaluated, using quantitative real-time polymerase chain reaction (qPCR), respectively immunohistochemistry (IHC), on post-TURBT tissue specimens, from before (pre-) and after (post-) complete spontaneous bladder tumor necrosis under Nivolumab.

## 2. Materials and Methods

### 2.1. Conceptual Design and Study Cohort

During clinical practice, we were faced with the very unique and somewhat peculiar situation of a patient diagnosed with advanced MM, undergoing second-line oncological treatment (BRAF/MEK inhibitors) for recurrence, which thereafter developed an additional high-risk NMIBC concurrently, managed conservatively initially, through repeated TURBTs. However, after multiple BC recurrences, the patient was to undergo a radical cystectomy; yet, due to his MM progression, immunotherapy with Nivolumab was administered beforehand for almost 5 months, i.e., both as an MM progression treatment and, coincidentally, as a neoadjuvant for BC. Ultimately, upon admission for elective cystectomy, preoperative cystoscopy demonstrated complete spontaneous necrosis of the large, multiple, high-risk bladder tumors under Nivolumab. Therefore, an organ-sparing strategy was reconsidered. Despite this initial unexpected response, BC recurrence occurred during the following months, requiring resection and, ultimately, a salvage cystectomy due to uncontrollable hematuria.

To contextualize the uniqueness of this case, we conducted a comprehensive ten-year retrospective review of bladder cancer cases at our institution (the Urology Department of the Timisoara County Emergency Hospital), which manages approximately 150 cystectomies and 250 transurethral resections annually. Our search specifically focused on identifying cases where concurrent active neoplasia influenced BC evolution. Despite examining over 2000 cases, no comparable situations were identified, confirming the exceptional nature of this case and supporting our decision to proceed with this single-case analysis.

This unique clinical scenario offered a great research opportunity, as this unusual evolution of the BC seemingly implicated the BRAF pathway in the oncogenesis of BC, while also offering the perfect platform for the analysis of PD-L1 IHC as a predictor of therapeutic response to ICIs in BC. Herein, we established our two main research objectives:

1. The molecular evaluation of somatic *BRAF* gene mutations in the concurrently occurring bladder tumors, i.e., occurring under treatment with BRAF/MEK inhibitors for V600E positive, recurrent MM, as possible emerging drivers of carcinogenesis in BC;
2. The assessment of PD-L1 immuno-expression in BC tissue as a predictor of therapeutic response to ICIs.

After obtaining the required ethical approvals, all relevant clinical data regarding this multiple concomitant neoplasia patient was collected and coherently organized to reflect the study’s clinical background accurately. To this end, hospital records and all other available medical data (imaging, pathology reports, therapeutic protocols) were assessed in order to document the overall clinical context and precise oncological evolution. After carefully analyzing the patient’s concurrent BC evolution, we collected the available biological material (post-TURBT, formalin-fixed paraffin-embedded (FFPE), tumor specimens), corresponding to the two key moments we identified as being most relevant for our research goals, namely: (1) before experiencing complete spontaneous bladder tumor necrosis; (2) upon recurrence, postspontaneous necrosis.

After preliminary sampling and subsequent histological processing of the acquired BC biological material, conventional microscopy was used to assess the resulting hematoxylin-eosin (HE) stained bladder tumor sample slides. Pathological diagnosis and staging were reconfirmed, and key morphological traits were documented.

Targeted IHC staining sample selection was achieved by individually assessing the multiple HE slides obtained by repeated sampling of each individual paraffin block, corresponding to pre- and postspontaneous necrosis tumor tissue specimen study populations, for inclusion criteria conformity, i.e., the paraffin block sections with carcinomatous tissue must contain over 100 viable tumor cells per section to be relevant. Herein, due to the limited biological material available, only 20 sections could be obtained, 10/block, which met these pre-established inclusion criteria for further IHC staining. Furthermore, the pre- and postspontaneous necrosis samples were additionally sampled for qPCR detection of *BRAF* gene mutations. Sample inclusion criteria for molecular assessment mandated the presence of sufficient tumor material in excision samples (up to 2.00 mm^3^) to obtain adequate deoxyribonucleic acid (DNA) extraction, with an adequate genomic DNA (gDNA) sample concentration (i.e., within an input range of 10–80 ng/μL, with a total amount of gDNA within 50–400 ng/μL per reaction).

### 2.2. PD-L1-Targeted Immunohistochemistry Expression Analysis

Within this current IHC study, the biological material available was limited to a single block of FFPE BC tissue per each TURBT, pre- and postspontaneous necrosis. We began processing these specimens by first sectioning them using a microtome to obtain multiple preliminary 3 μm thick sections per block. These preliminary tissue sections were then transposed onto histological glass slides (silanized, albumin pre-treated), and distilled water (one drop/slide) was added to aid in repositioning and prevent artifacts from appearing. Following excess fluid removal, these slides underwent thermal treatment (58 °C, for 30 min), then deparaffination (benzene, 58 °C, ≥30 min). Finally, the preliminary BC tissue slides were stained with hematoxylin-eosin (HE) using a well-established, automated platform (Leica Autostainer XL system, Leica Biosystem Newcastle Ltd., Newcastle, UK), then reassessed morphologically using conventional photon microscopy (Nikon E600, Nikon, Tokyo, Japan) to reconfirm diagnosis and staging and to determine the samples’ eligibility for PD-L1 IHC.

#### 2.2.1. Tissue Preparation and Staining Protocol

For PD-L1 immuno-expression analysis, we used the Dako PD-L1 IHC 22C3 pharmDx kit on FFPE BC tissue sections. This assay employs a monoclonal mouse anti-PD-L1 antibody (Clone 22C3) specifically designed for FFPE tissue sections. The staining was performed using the Dako Autostainer Link 48, an automated staining system, alongside the EnVision FLEX visualization system (Agilent Technologies, Inc., Santa Clara, CA, USA). The entire procedure was carried out following the manufacturer’s protocol to ensure optimal performance, consistency, and reproducibility of results.

Thus, additional sections were achieved through slicing for both blocks (10/block) and then were mounted onto Dako FLEX IHC microscope slides, which were then placed in a 60 °C oven for at least one hour to ensure adherence. The mounted sections underwent a deparaffinization process using xylene to remove paraffin, followed by rehydration through graded alcohol baths (100%, 95%, 70% ethanol) and distilled water.

Antigen retrieval was performed in EnVision FLEX Target Retrieval Solution (Low pH, 50×) using the Dako PT Link module (Agilent Technologies, Inc., Santa Clara, CA, USA). The retrieval solution was preheated to 65 °C, and the slides were incubated at 97 °C for 20 min to unmask the epitopes. After antigen retrieval, the sections were cooled to 65 °C and then washed in EnVision FLEX Wash Buffer (Agilent Technologies, Inc., Santa Clara, CA, USA) to remove any residual retrieval solution.

The IHC staining procedure was performed on the Dako Autostainer Link 48, an automated system that ensures consistency in reagent application and timing. The tissue sections were first treated with a peroxidase-blocking reagent for 5 min to block endogenous peroxidase activity, reducing nonspecific staining. Following blocking, the primary antibody, monoclonal mouse anti-PD-L1 (Clone 22C3), was applied at a concentration of approximately 3 μg/mL and incubated for 20 min at room temperature to allow binding to PD-L1 epitopes present in the tumor tissue. After primary antibody incubation, the slides were washed and treated with a mouse linker antibody for 15 min, followed by the application of the EnVision FLEX Visualization Reagent-HRP, which contains dextran polymers coupled with horseradish peroxidase. This reagent was incubated for 20 min to amplify the signal from the primary antibody. The chromogenic detection step was carried out using DAB+ substrate-chromogen solution, resulting in a brown precipitate at the site of antigen-antibody binding. The chromogenic reaction was monitored visually under the microscope to prevent overstaining.

The sections were then counterstained with hematoxylin for 5 min to provide a contrast, allowing for easier identification of cellular structures. After counterstaining, the slides were rinsed in running tap water, dehydrated through graded alcohols, cleared in xylene, and mounted within a permanent mounting medium. Care was taken to ensure that tissue sections did not dry out during the staining process, as this could lead to artifacts.

#### 2.2.2. Interpretation and Scoring

The IHC slides were examined microscopically at magnifications ranging from 100× to 400×. The assessment was performed on the entire tissue section, with a minimum requirement of 100 viable tumor cells for the evaluation to be considered adequate.

To objectively quantify PD-L1 expression, the combined positive score (CPS) was calculated according to standard protocols by determining the ratio between PD-L1-positive cells (including viable tumor cells and/or tumor-associated mononuclear inflammatory cells such as lymphocytes and macrophages) and the total number of viable tumor cells (both PD-L1-positive and negative), multiplied by 100.

$$\mathrm{CPS}=\frac{\left(PD-L1+\right)tumor\;cells+(PD-L1+)tumor-associated\;immune\;cells}{total\;viable\;tumor\;cells(PD-L1+/-)}\times 100$$

Positive PD-L1 staining was defined as distinct complete and/or partial membrane staining of any intensity in viable tumor cells, whereas, for tumor-associated mononuclear inflammatory cells, both membrane and/or cytoplasmic staining of any intensity were deemed acceptable for positivity. Specimens were categorized as PD-L1 negative if CPS < 10 and PD-L1 positive if CPS ≥ 10. All specimens contained an adequate number of viable tumor cells (>100 cells per section) for evaluation.

#### 2.2.3. Quality Control

Stringent quality control measures were implemented to validate the IHC staining procedure. Control slides were included in each staining run, containing both PD-L1 positive and negative cell lines. Specifically, NCI-H226 cells, which exhibit moderate PD-L1 expression, and MCF-7 cells, which are PD-L1 negative, were used as controls. Positive control slides were stained to confirm the effectiveness of the staining reagents and protocol, while negative control slides were used to verify the absence of nonspecific staining.

Each control slide was evaluated prior to the interpretation of patient samples. The NCI-H226 cell line, which served as the positive control, demonstrated appropriate membrane staining in over 70% of cells at an intensity of moderate or greater, whereas the MCF-7 negative control exhibited no specific staining, ensuring that the staining system was functioning correctly. Additionally, internal negative controls within the patient tissue, such as areas without tumor or immune infiltration, were also examined to verify the specificity of staining.

The use of appropriate positive and negative controls ensures that the results obtained from patient tissues are reliable. Any deviations in staining patterns in the control slides prompted a re-evaluation of the staining run to identify potential procedural issues. Regular equipment maintenance and reagent quality checks were also conducted to ensure consistent assay performance throughout the study.

### 2.3. BRAF Gene Mutation Analysis

Detection of *BRAF* gene mutations was carried out using the gb ONCO BRAF (V600E) diagnostic kit (Genbox, Montpellier, France), a CE IVD-marked assay validated for in vitro diagnostics. This ready-to-use kit employs qPCR, with fluorescently labeled probes and allele-specific primers, to identify and quantify specific *BRAF* gene mutations, i.e., a range of nine established *BRAF* mutations commonly contributing to the oncogenesis of various cancers. The assay’s sensitivity allows for a limit of detection (LOD) of 0.05% mutated *BRAF* alleles within a wild-type (WT) background at a minimum DNA input of 100,000 copies per reaction.

#### 2.3.1. Tissue Preparation and DNA Extraction

For both pre- and postnecrosis FFPE tumor tissue specimens, a single thicker section was obtained (10 µm) and microdissected, to achieve >80% tumor tissue on the final sample, then placed into a 1.5 mL microcentrifuge tube. Thereafter, gDNA extraction was conducted from the processed tissue sections following the manufacturer’s protocols for FFPE samples (Maxwell RSC DNA FFPE Kit, Promega Corporation, Madison, WI, USA). Importantly, for each sample, FFPE sections were mandated to amount to a maximum total input volume of 2.0 mm^3^, as per the manufacturer’s recommendations.

After extraction, DNA concentration was quantified in each sample, using spectrophotometry (i.e., Nanodrop spectrophotometry, requiring an A260/280 ratio between 1.8–2.0) in order to ensure compliance with the gb ONCO BRAF (V600E) diagnostic kit’s recommended input range of 10–80 ng/μL, i.e., with a total amount of gDNA within 50–400 ng/μL per reaction, and, implicitly, to meet the pre-established inclusion criteria for genomic analysis.

#### 2.3.2. Mutation Detection Protocol

The specific *BRAF* gene mutations detected by the gb ONCO BRAF (V600E) diagnostic kit include the following:

1. p.V600A (c.1799T>C): Thymine replaced by cytosine at position 1799 of the coding DNA sequence (CDS), the substitution of valine (V) with alanine (A) at codon 600.
2. p.V600D (c.1799_1800delTGinsAT): Deletion of thymine and guanine at positions 1799 and 1800 of the CDS, replaced by adenine and thymine, leading to a valine (V) to aspartic acid (D) substitution at codon 600.
3. p.V600E (c.1799T>A): Thymine to adenine substitution at position 1799 of the CDS, resulting in valine (V) being replaced by glutamic acid (E) at codon 600.
4. p.V600E (c.1799_1800delTGinsAA): Deletion of thymine and guanine at positions 1799 and 1800 of the CDS, replaced by two adenines, causing a valine (V) to glutamic acid (E) substitution at codon 600.
5. p.V600G (c.1799T>G): Thymine to guanine substitution at position 1799 of the CDS, leading to valine (V) being replaced by glycine (G) at codon 600.
6. p.V600K (c.1798_1799delGT>insAA): Deletion of guanine and thymine at positions 1798 and 1799 of the CDS, replaced by two adenines, resulting in valine (V) being substituted by lysine (K) at codon 600.
7. p.V600M (c.1798G>A): Guanine to adenine substitution at position 1798 of the CDS, causing valine (V) to be replaced by methionine (M) at codon 600.
8. p.V600R (c.1798_1799delGT>insAG): Deletion of guanine and thymine at positions 1798 and 1799 of the CDS, replaced by adenine and guanine, leading to valine (V) being substituted by arginine (R) at codon 600.
9. p.K601E (c.1801A>G): Adenine to guanine substitution at position 1801 of the CDS, resulting in lysine (K) being replaced by glutamic acid (E) at codon 601.

These mutations are located within the kinase domain of the BRAF protein and are known to activate the MAP kinase/ERK-signaling pathway, contributing to oncogenesis in various cancers. Accurate detection of these mutations is essential for guiding targeted therapies and informing clinical decision-making.

Thus, the extracted DNA underwent qPCR amplification, with allele-specific primers designed to amplify the *BRAF* gene region containing codon 600 and adjacent sequences, and hydrolyzing probes, fluorescently labeled by FAM fluorophore (λ_EXCITATION_ = 495 nm; λ_EMISSION_ = 520 nm). Herein, the gb ONCO BRAF (V600E) diagnostic kit comprises the following reagents:

1. Assay qPCR BRAF Control V600E (3.125× concentration)
2. Assay qPCR BRAF MUT V600E (3.125× concentration)
3. Master Mix BRAF V600E (2.083× concentration)
4. Standard WT BRAF V600E (10^4^ copies/µL)
5. Standard MUT 1% BRAF V600E (10^4^ copies/µL)
6. Deionized Water

Target sequences were amplified starting from extracted gDNA using a Bio-Rad CFX96 thermocycler (Bio-Rad Laboratories, Hercules, CA, USA), previously validated for use with this diagnostic kit.

For each sample, we prepared two separate reaction mixes: one for the control assay and one for the mutation assay. Each qPCR reaction was performed in a total volume of 25 μL, which included the following: 12 μL of Master Mix BRAF V600E; 8 μL of either Assay qPCR BRAF Control V600E or Assay qPCR BRAF MUT V600E; and respectively, 5 μL of gDNA template. For each sample, both reactions were triplicated. Quality controls were included: (1) positive control—using Standard MUT 1% BRAF V600E as the template; (2) negative control—using Standard WT BRAF V600E as the template; (3) no template control (NTC)—using deionized water instead of gDNA.

Thermal cycling was conducted according to the kit’s recommended protocol:

7. Initial Denaturation: 95 °C for 3 min.
8. Denaturation: 95 °C for 10 s (50 cycles).
9. Annealing + Elongation (with fluorescence acquisition): 60 °C for 20 s (50 cycles).

Fluorescence data were collected at the end of each annealing + elongation step using the FAM/SYBR detection channel. Resulting amplification curves were generated and analyzed through the system software to determine cycle threshold (Ct) values for each reaction.

#### 2.3.3. Data Interpretation and Quality Controls

After qPCR completion, Ct values for each sample are obtained separately from the MUT assay (Ct_M_) and the Control/WT assay (Ct_C_). The kit specifies using these Ct values to compute a Fold Change (FC) that accounts for assay-specific correction factors. Herein, FC = (Ct_M_ − Ct_C_) − 2.37, where 2.37 is a fixed kit-defined offset to align the FC distribution around zero for WT samples. Lower FC values (i.e., negative or small positive) suggest the presence of mutant alleles, whereas higher FC values are consistent with WT genotypes.

Regarding cutoff values and quality controls, the manufacturer-provided standard WT BRAF V600E is used to confirm assay specificity for the WT sequence. Its FC value typically falls above 9.1 when tested under correct reaction conditions. If the WT standard’s FC < 9.1, the run is considered invalid or the reaction conditions suboptimal, necessitating re-analysis. Conversely, the standard MUT 1% BRAF V600E must generate an FC value in the 5.9–8.1 range to confirm assay sensitivity at the 1% mutant allele fraction (LOD~0.27%). If the 1% MUT standard’s FC deviates significantly from this interval, the run may not be reliable, and re-analysis is recommended. Lastly, NTC wells must show no amplification (i.e., baseline fluorescence only). Any detectable signal in NTC indicates possible reagent or carryover contamination, invalidating the run.

Kit guidelines provide two main criteria when interpreting sample FC values:

1. FC vs. cutoff:
  - Each run has an assigned cutoff FC value (often near 9–10, as adjusted by the WT standard).
  - If a sample’s FC exceeds the cutoff FC, the sample is classified as WT.
  - If a sample’s FC falls below the cutoff FC, the sample is considered mutated (provided the second criterion below is also met).
2. ΔCt Confirmation:
  - The difference (CtMSAMPLE − CtCWT-STANDARD) must be <13 cycles to confirm that the sample’s signal is truly within the quantifiable range of the assay.
  - Samples with FC < cutoff, but (CtMSAMPLE − CtCWT-STANDARD) ≥ 13 are deemed below the LOD and thus effectively classified as WT (or unquantifiable mutation).

For samples meeting both mutation criteria (FC < cutoff and ΔCt < 13), the kit describes an approximate calculation for the percentage of mutant alleles:

  - Mutant allele fraction (%) = 100 × 1.93 − FC.

This formula correlates the FC value with the mutant allele percentage. Samples with increasingly negative FC values yield higher estimated mutation burdens. Note, however, that this calculation does not account for total input DNA variation and should be treated as semiquantitative.

In conclusion, runs were declared valid only if the WT and 1% MUT standards fell within their specified FC ranges and the NTCs remained negative. Thus, a sample was interpreted as mutated only if FC < cut-ff FC and ΔCt < 13 cycles. Conversely, a sample was considered WT if FC > cutoff FC or if ΔCt ≥ 13 places it below the detection threshold.

## 3. Results

### 3.1. Clinical Background

**Figure 1:**
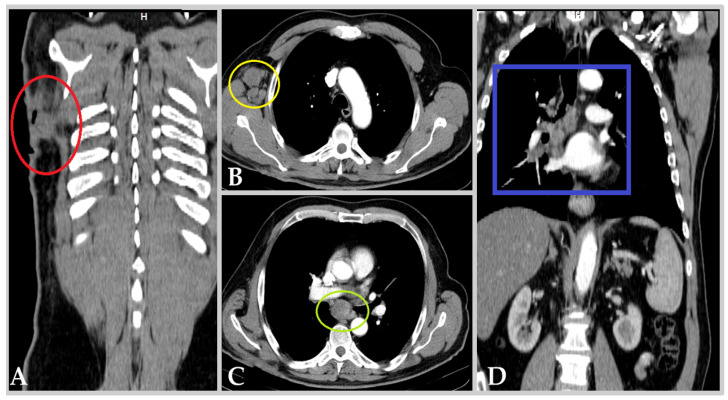
Imaging at initial presentation, i.e., details from contrast-enhanced computer tomography of thorax and abdomen, arterial phase, demonstrating malignant melanoma clinical stage at diagnosis (pT4b cN2 M1lym): (**A**) coronal view, primary lesion (red circle) in the right subscapular area; (**B**) axial view, right axillary adenopathic block (yellow circle); (**C**) axial view, large upper mediastinal adenopathy (green circle); (**D**) coronal view, right hilar and peribronchial adenopathic block (blue square).

The patient, a 68-year-old male, nonsmoker, without any known relevant environmental exposure to carcinogens, with diabetes mellitus type 2 controlled through diet, initially presented with an extensive, 12/12 cm, heterogeneous and hyper-pigmented skin lesion, localized in the right subscapular area, complicated with an adjacent 5/5 cm purulent collection, exteriorized through a visible cutaneous fistula. Further imaging and clinical assessment were suggestive of advanced malignant melanoma (MM) of the posterior thorax (see Figure 1A), metastasized to the right axillary (see Figure 1B), mediastinal (see Figure 1C), and right hilar/peribronchial lymph nodes (see Figure 1D). Thus, he was diagnosed in 2014 with MM stage IVa (pT4b cN2 M1lym), for which he underwent primary surgical excision and right axillary lymphadenectomy (pN1) with adjuvant Dacarbazine chemotherapy. This strategy proved initially effective, granting the patient 4 years of disease-free survival. However, in 2019, he experienced pulmonary and mediastinal lymph node relapse, confirmed as BRAF-V600E-positive through excisional biopsy. Consequently, the first line of targeted combined chemotherapy with Dabrafenib and Trametinib (BRAF/MEK inhibitors) was initiated on 9 May 2019.

Before the administration of BRAF/MEK inhibitors, as per oncological recommendations, a pretherapeutic urological consultation was obtained. Ultrasound assessment documented a normal bladder, with an apparently regular contour and no evidence of endovesical tumor growth. Conversely, after 2 months of anti-BRAF/MEK therapy, macroscopic hematuria ensued, only to further reveal an apparently “de novo” bladder tumor, ~2.8 cm in diameter, localized on the right lateral wall. TURBT was performed, and pathology indicated high-grade papillary urothelial carcinoma pTaG2, with the caveat of absent muscularis propria in the resection specimens (see Table 1). Consequently, anti-BRAF/MEK therapy was temporarily discontinued, as per oncological protocols.

Endoscopic reassessment at six weeks post-TURBT revealed an epithelialized tumor resection base, which was then biopsied, and pathological evaluation did not show any signs of tumor infiltration. Thus, BRAF/MEK inhibitors were reinstated on 1 October 2019, approximately five weeks after endoscopic bladder reassessment, i.e., after a total of ~11 weeks off of anti-BRAF/MEK therapy. Thereafter, at 3 months post reinitialization of combined targeted chemotherapy, contrast-enhanced computer tomography (CECT) documented MM response (stationary pulmonary nodules and reduced mediastinal adenopathy), while cystoscopy confirmed normal bladder morphology without BC recurrence.

**Figure 2:**
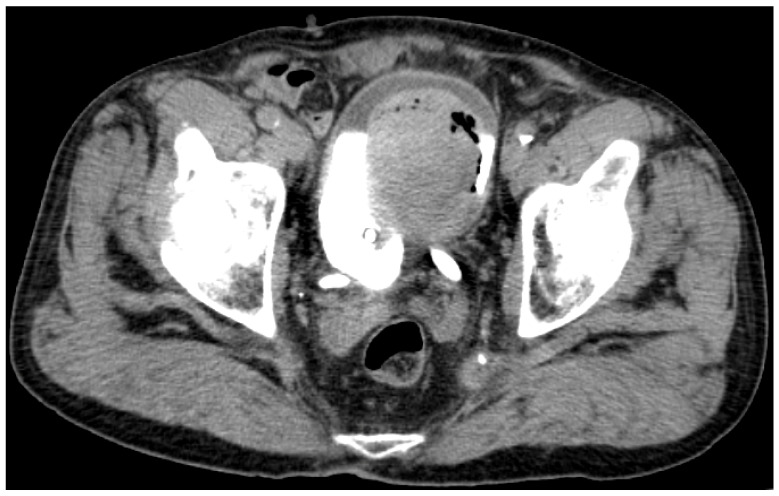
Contrast-enhanced computer tomography scan of the pelvis, axial view, and excretory phase (15 min), showing a voluminous recurrence of the bladder tumor during combined targeted chemotherapy (anti-BRAF/MEK) for malignant melanoma.

Unfortunately, after one year of apparent MM remission, a second episode of macroscopic hematuria ensued and mandated imaging reevaluation. A whole-body CECT scan demonstrated MM progression, with additional pulmonary lesions, progression of existing lesions, mediastinal lymphatic progression, and, additionally, a voluminous recurrence of the bladder tumor (see Figure 2). Consequently, anti-BRAF/MEK treatment was permanently discontinued, and the second-line treatment with Nivolumab (anti-PD-1 antibody) was initiated later (1 September 2020).

The TURBT of this recurrence, on 2 June 2020, revealed multiple large endovesical tumor formations at the level of the bladder trigone, left lateral wall, right lateral wall, and anterior wall, with generalized wall friability. A biopsy-targeted resection was performed, and histopathological examination described high-grade papillary urothelial carcinoma (pTa G2), with the presence of muscularis propria in the sample but with extensive residual tumor tissue remaining unresected (see Table 1).

A cystoscopy performed on 6 July 2020 revealed approximately 50% of the bladder occupied by tumor formations. Due to the high volume of the tumor and the persistent associated episodes of hematuria, a secondary biopsy was required for adequate staging. The subsequent restaging resection pathologically confirmed a high-grade urothelial carcinoma (pT1 G2), i.e., a high-risk bladder tumor (see Table 1). This prompted the recommendation for radical cystectomy, per the current guidelines of the European Association of Urology (EAU).

After one month of concomitant immunotherapy, on 30 September 2020, the patient was admitted for elective radical cystectomy. However, during preoperative workup, ultrasound and cystoscopy evaluations revealed complete spontaneous tumor necrosis (see Table 1). Thus, immunotherapy was continued, and an organ-preservation strategy was recommended for the urothelial carcinoma, now in complete remission macroscopically.

Three months later, during tomographic reevaluation, MM remission was observed; however, a bladder tumor recurrence was detected once more. The histopathological analysis from 1 February 2021 described a high-grade papillary urothelial carcinoma (pT1 G2) with focal squamous features and absence of muscularis propria (see Table 1).

Within seven weeks post-TURBT, a salvage radical cystectomy, with cutaneous ureterostomy, was performed due to recurrent, persistent macroscopic hematuria, which proved impossible to control endoscopically. Histopathological analysis of the cystectomy specimen (23 March 2021) revealed early urothelial carcinoma (pTa G2), with 34 lymph nodes collected during bilateral ilio-obturator lymphadenectomy, free of metastases (pN0) and clean surgical margins (R0). Additionally, an incidental prostatic adenocarcinoma (Gleason 3 + 3 = 6, pT2a, OMS/ISUP I/V) was also discovered (see Table 1).

During follow-up, prostate-specific antigen (PSA) was consistently <0.004 ng/mL and no additional urothelial carcinoma recurrences were seen. Moreover, to date, as documented by the latest imaging available (11 November 2024), the MM has shown consistent remission under Nivolumab; yet, a circumferential parietal thickening of the rectum has now been newly documented with the patient currently awaiting colonoscopy.

All in all, we observed the occurrence of urothelial bladder carcinoma, concurrent with the initiation of anti-BRAF/MEK chemotherapy, and its progression under this treatment, suggesting a possible causal role for BRAF/MEK inhibitors in the carcinogenesis of urothelial carcinoma, followed by complete spontaneous tumor necrosis under Nivolumab, and then tumor recurrence post-necrosis, under the same immunological treatment, implicitly claiming the establishment of some occult mechanism of resistance to immunotherapy.

### 3.2. Prespontaneous Bladder Tumor Necrosis Tissue Samples

**Figure 3:**
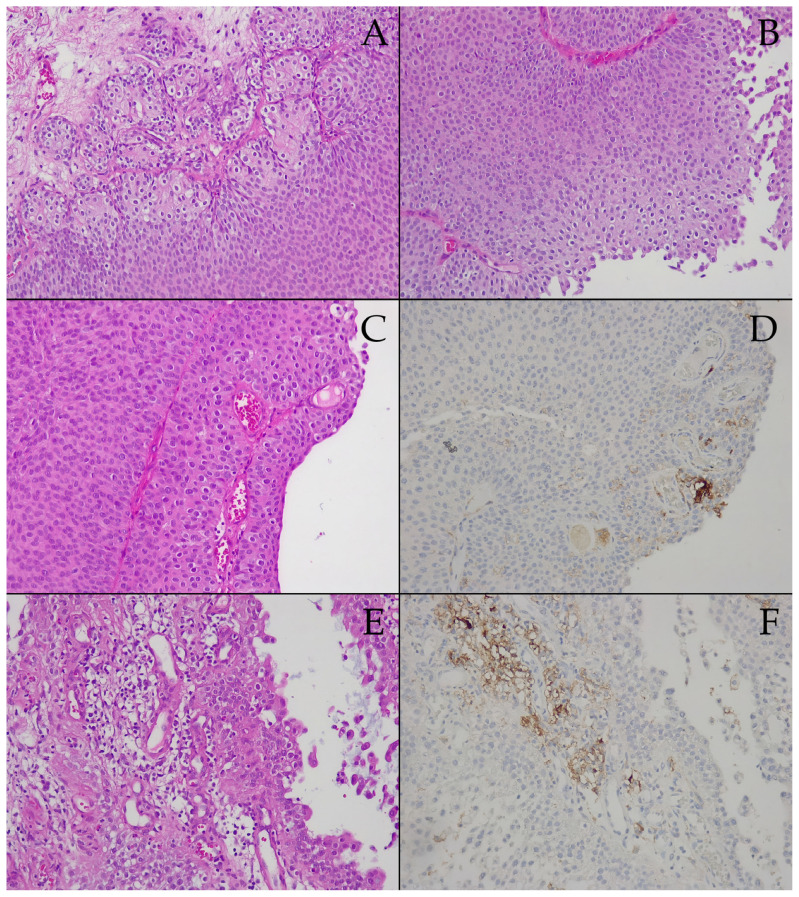
Microscopic findings in prespontaneous bladder tumor necrosis tissue fragments obtained through transurethral resection (biopsy): (**A**) 200×, HE, nests of neoplastic urothelial cells in the deep portion of the proliferation, showing focal invasion of the submucosa; (**B**) 200×, HE, high-grade traits, G2 cellularity, in the superficial portion of the proliferation, with some mitotic activity; (**C**) 200×, HE, intratumoral vascular elements in the superficial portion of the proliferation; (**D**) 200×, IHC with anti-PD-L1 (Dako clone 22C3), moderate to intense staining reaction in tumor cells, and isolated infiltrating immune cells (CPS = 5%); (**E**) 200×, HE, high-grade area with abundant vascularity and tumor-associated inflammatory cells; (**F**) 200×, IHC with anti-PD-L1 (Dako clone 22C3), moderate to intense staining reaction in tumor cells and tumor-associated inflammatory cells (CPS = 5%).

The tissue specimens examined within this subgroup were resected as a biopsy sample, with a large amount of residual tumor; TURBT aims to validate the indication for radical cystectomy rather than act curatively. Herein, pathology assessment could only demonstrate pT1, namely urothelial carcinoma focally invading submucosa (see Figure 3A), with negative muscularis propria, yet still, high-grade G2 (see Figure 3A,B), which alongside the large tumor volume, constitute sufficient rationale to recommend radical cystectomy, especially within the aforementioned clinical context.

In the superficial portion of the proliferation (see Figure 3C), intratumoral vascular elements are consistently seen (see Figure 3C,E). Regarding PD-L1 IHC, the PD-L1 IHC images (Figure 3D,F) were chosen to mirror their HE counterparts in the first column of Figure 3C,E. We report that isolated groups of neoplastic urothelial cells, alongside tumor-associated inflammatory cells, showed moderate to intense staining reactions (see Figure 3D,F); yet overall, PD-L1-expression manifested a CPS of only five, i.e., prespontaneous necrosis tumor tissue samples were labeled negative for PD-L1. Conversely, the *BRAF* gene mutations included in our panel were negative in the prespontaneous necrosis BC tissue specimens.

### 3.3. Postspontaneous Bladder Tumor Necrosis Tissue Samples

**Figure 4:**
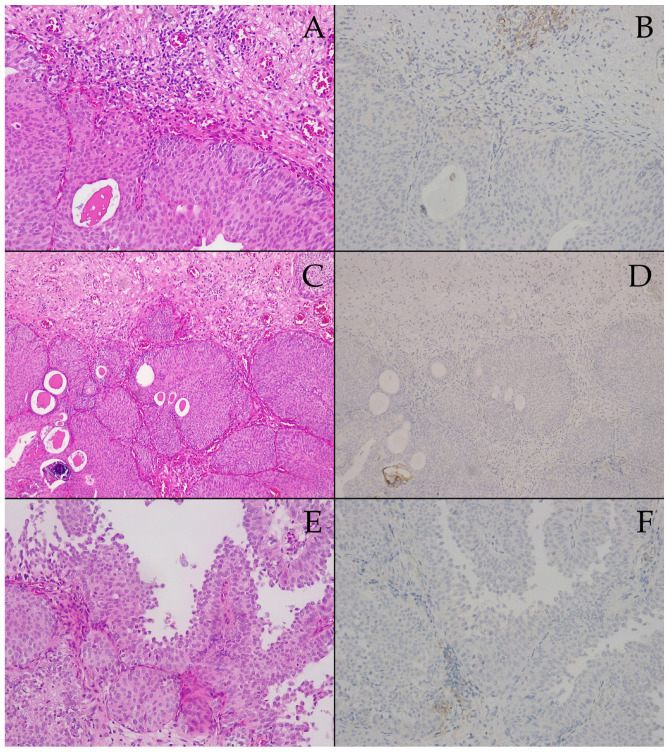
Microscopic findings in postspontaneous bladder tumor necrosis relapse tissue fragments obtained through transurethral resection: (**A**) 200×, HE, urothelial carcinoma proliferation, invading the basal membrane and superficial submucosa focally, while associating a significant submucosal desmoplastic reaction; (**B**) 200×, IHC with anti-PD-L1 (Dako clone 22C3), a moderate color reaction in tumor-associated inflammatory cells (CPS = 1); (**C**) 200×, HE, urothelial carcinoma proliferation, with abundant vascularization and a microcalcification focus; (**D**) 200×, IHC with anti-PD-L1 (Dako clone 22C3), a very weak color reaction in rare tumor-associated inflammatory cells (CPS = 1); (**E**) 200×, HE, urothelial carcinoma with a classic papillary-type growth pattern; (**F**) 200×, IHC with anti-PD-L1 (Dako clone 22C3), a weak color reaction in scarce tumor cells and a few tumor-associated inflammatory cells (CPS = 1).

The tissue specimens examined within this subgroup were resected with curative intent, yet TURBT was incapable of obtaining muscularis proria samples. Thus, pathology assessment staged the tumor as minimum pT1, with isolated small groups of urothelial carcinoma cells focally invading the basal membrane and superficial submucosa, which would, at times, demonstrate desmoplastic reactions (see Figure 4A). As opposed to the prespontaneous necrosis subgroup, rare squamous morphological traits and isolated microcalcifications were encountered (see Figure 4C), yet the papillary architecture remained prevalent (see Figure 4E). Abundant intratumoral vascular elements are consistently seen here as well (see Figure 4A,C). Cellularity was still high-grade G2 (see Figure 3A,C), yet the previous unexpected results with conservative treatment allowed for a relaxed attitude towards radical surgical intervention.

The PD-L1 IHC images (Figure 4B,D,F) were chosen to mirror their HE counterparts in the first column of Figure 4A,C,E. Despite some isolated moderate reactions encountered in tumor-associated inflammatory cells (see Figure 4B), for the most part, reactivity was either weak (see Figure 4F) or very weak (see Figure 4D), scarcely seen in immune infiltrates, and exceptionally in tumor cells (see Figure 4F). Overall, PD-L1 expression was decreased even further, with the CPS = 1, i.e., postspontaneous necrosis tumor tissue samples were also labeled negative for PD-L1. Similarly, the *BRAF* gene mutations included in our panel were negative in the postspontaneous necrosis BC tissue specimens.

## 4. Discussion

### 4.1. Clinical Considerations

Morphopathological evaluation of post-TURBT specimens provides critical information about parietal invasiveness and histopathological variants, with decisive implications for therapeutic management. Clinical staging involves assessing tumor extension before radical cystectomy through post-TURBT pT staging, bimanual examination, liver function tests (i.e., hepatic metastases), chest radiography (at least), and (thoracic-)abdominopelvic CT urography (cNM) [12,27]. Contemporary studies report pathological upstaging from clinical staging in 50% of cases and downstaging in 25% of cases for pT2 disease, after radical cystectomy [28].

Despite aggressive therapeutic interventions, of the 20–30% of BC patients who present with MIBC at initial evaluation, a significant proportion will experience recurrence and ultimately succumb to this neoplastic pathology [29]. Clinical understaging is relatively frequent at initial MIBC diagnosis, necessitating a multimodal, interdisciplinary approach to properly integrate and individually personalize available therapeutic options (surgery, systemic chemotherapy, and radiotherapy).

MIBC cases predominantly comprise those diagnosed with the infiltrative stage at initial presentation, with a subpopulation (approximately 20%) representing patients initially diagnosed with nonmuscle-invasive tumors, that subsequently progress to infiltrative disease. MIBC represents a lethal clinical entity associated with a cancer-specific mortality of 85% at 2 years from diagnosis if left untreated [30].

Additionally, some studies report less favorable prognosis in cases that progress from NMIBC, though these data may be partially attributed to the initial understaging of presumed NMIBC cases that were actually MIBC from the start and/or significant progression under conservative treatment rather than necessarily reflecting a significant difference in tumor biological aggressiveness [31]. Nonlocoregional recurrences after conservative treatment reflect the presence of micrometastatic disease at diagnosis, diminishing the survival rate of MIBC patients [31,32]. The significant risk of micrometastatic disease presence and the current inability to accurately identify patients with nonorgan-limited cancer before definitive local therapy (TURBT) continues to complicate therapeutic decisions and advocates for a multidisciplinary therapeutic approach.

Conversely, TURBT represents the gold standard diagnostic method for BC and implicitly for MIBC. Although data is limited on this topic, complete resection of macroscopically visible tumor tissue is generally recommended if feasible and safely achievable. Complete resection reduces local tumor burden, potentially optimizing response to neoadjuvant chemotherapy and increasing success rates in organ-preservation therapeutic strategies [33]. Additionally, prospective randomized trials of neoadjuvant chemotherapy have unequivocally demonstrated that pT0 status at radical cystectomy carries significantly improved survival, which holds true even for patients who did not receive neoadjuvant chemotherapy, suggesting a potential benefit from aggressive resection [34]. However, “heroic” resections, especially in cases with macroscopic appearance suggestive of advanced, invasive forms, carry risks of perforation, bleeding, and massive hematogenous dissemination [35], thus emphasizing that complete resection should only be pursued when feasible and safely achievable.

### 4.2. Molecular Oncogenesis and BRAF Gene Mutations Assessment

BC represents maybe the most genetically heterogeneous urogenital neoplasm, being one of the most frequently mutated human cancers, preceded in mutation rates only by lung and skin cancer [36,37]. Amongst these mutations, most frequently encountered are mutations involving the promoter of the gene encoding telomerase reverse transcriptase (*TERT*), seen in 70–80% of patients with BC [38,39]. As a whole, the genetic alterations seen in BC fall into two main categories:

- Mutations mostly unrelated to clinical subtype—changes in chromosome 9 and/or the *RAS* family of proto-oncogenes (H-, K-, and N-RAS);
- Mutations related to specific grades/stages of the disease—fibroblast growth factor receptor 3 (*FGFR3*) mutations for low-grade noninvasive papillary urothelial BC; *TP53* and *RB1* alterations in muscle-invasive disease [40].

More recently, the TCGA Research Network has identified several additional mutated genes involved in cell cycle regulation (*CDKN1A*), nucleotide excision repair (*ERCC2*), and retinoic acid-mediated gene activation (*RXRA*), which had not been previously incriminated as significant in BC tumorigenesis [41]. The large volume of resources invested in the molecular characterization of BC pathology is expected to improve the accuracy of BC diagnosis and risk stratification while allowing for the development of novel therapeutic approaches, as well as new urine/blood biomarkers for disease surveillance, useful in early detection and disease monitoring following treatments [39].

Given this complex molecular landscape, our study retrospectively evaluated *BRAF* gene mutations and PD-L1 expression, using qPCR and IHC, respectively, in a unique clinical setting: a high-risk bladder tumor arising during anti-BRAF/MEK therapy for concurrent MM, followed by unexpected complete spontaneous necrosis of the bladder tumor under Nivolumab immunotherapy, and subsequent relapse. Within the current work, our evaluations were carried out on transurethral resection specimens, both before completing spontaneous tumor necrosis (P1) and after this event (P2).

*BRAF* mutations are well-known oncogenic drivers in MM but have only been sporadically reported in BC. In fact, to the best of our knowledge, only three cases have been described so far, each with a different mutation involved. Of these, *BRAF-V600E* was discovered in a case of conventional urothelial carcinoma pT1aG2, and *BRAF-V600L* in another case of conventional urothelial carcinoma pT2bG3 [42]. Another study highlights a rare *NRF1-BRAF* fusion variant in metastatic urothelial carcinoma of the renal pelvis, which confoundingly showed an initial therapeutic response to Trametinib [43].

To complicate matters further, therapeutic response to Trametinib—predominantly in basal-origin urothelial subtypes—has been demonstrated in urothelial carcinoma cell cultures through drug activity screening studies [44]. However, sequencing of these tumor cells revealed no mutations in the coding genes, only other mutations indirectly interacting with the signaling pathway [44]. In corroboration of these results and despite the aforementioned theoretical basis, our molecular assessment did not detect *BRAF* mutations in the tumor tissue specimens, neither in prespontaneous necrosis samples nor in those from the recurrence. However, we must note that only a limited number of *BRAF* mutations, i.e., nine of the most common oncogenetic mutations in the signaling pathway, were evaluated herein via qPCR.

Importantly, phase 3 studies for BRAF/MEK inhibitors report an incidence of 10% for cutaneous squamous neoplasms and 1% for noncutaneous neoplasms [45]. Thus, it appears that, within the evolution of the current case, a therapeutic principle targeting a documented oncogenetic metabolic component (the BRAF/MEK signaling pathway) has led to the emergence of a new neoplasm—an aggressive urothelial carcinoma with tendencies for local progression and morphological divergence (appearance of squamous components). The underlying molecular oncogenetic mechanism remains unclear and appears independent of the targeted metabolic pathway.

All in all, our molecular results suggest that BRAF/MEK inhibition could create an oncogenic environment in certain tissues, potentially via compensatory activation of alternative mitogenic pathways, such as FGFR3 or RAS-MAPK signaling, which are more commonly involved in BC oncogenesis. Future studies should explore whether BRAF/MEK inhibition predisposes to urothelial transformation via nongenomic effects or epigenetic modifications. These findings highlight the need for more rigorous oncological surveillance in patients receiving long-term BRAF/MEK inhibition therapy, particularly in the presence of unexplained hematuria or other urological symptoms.

Regarding the incidental prostate adenocarcinoma, in other investigations focused on the urinary tract, Cho et al. discovered a notable 10.2% incidence of *BRAF* codon 600 mutations in prostate adenocarcinomas [46]. Intriguingly, these findings diverge from the observations of Burger et al. [47], who had previously communicated the absence of *BRAF* mutations in a cohort of 79 prostatic adenocarcinomas. Moreover, when examining germ cell tumors, it is noteworthy that activating *BRAF* missense mutations were identified in 9% of nonseminomas, but were notably absent in seminomas [48]. Importantly, the role of *BRAF* mutations seems negligible in the context of renal cell tumors [49], testicular germ cell tumors [50], as well as cervical, endometrial, and ovarian carcinomas [51], as per existing postulations [17].

### 4.3. PD-L1 Expression and Treatment Response Prediction

Concerning the IHC assessment, PD-L1 expression is commonly used as a predictive biomarker for response to ICIs, such as Nivolumab. The CPS system presently plays a pivotal role in guiding the selection of patients for immunologic treatment, necessitating, at a minimum, a PD-L1 combined expression of no less than >5% [52]. In fact, a CPS ≥ 10 is generally considered the threshold for PD-L1 positivity and a marker of potential immunotherapy benefit. However, in our case, the prenecrosis tumor exhibited a CPS of 5, suggesting a low likelihood of response, yet the patient experienced complete spontaneous tumor necrosis under Nivolumab. Conversely, the recurrent tumor showed even lower PD-L1 expression (CPS = 1), which may indicate the development of immunotherapy resistance mechanisms despite the initial response.

Overall, this case presents a paradox: tumor regression under Nivolumab, despite low PD-L1 expression, followed by recurrence with further decreased PD-L1 levels. This finding challenges the conventional reliance on PD-L1 as the sole predictive biomarker for immunotherapy response. Alternative mechanisms could explain this unexpected response, including:

- Tumor mutational burden (TMB)/neoantigen load: High TMB is associated with better response to ICIs, independent of PD-L1 status. Further genomic analysis would be required to determine whether these bladder tumors harbored a high TMB.
- Pre-existing immune activation: The prior anti-BRAF/MEK therapy may have primed the immune system, indirectly enhancing Nivolumab’s effectiveness. On the other hand, in correlation with the data previously presented, hinting at the possible pro-oncogenic role of BRAF/MEK inhibitors in BC, it may even be the case that, in fact, the interruption of anti-BRAF/MEK therapy was the real underlying cause of regression, not the initiation of immunotherapy.
- Microenvironment factors: The tumor’s immune microenvironment, including tumor-infiltrating lymphocytes and cytokine expression, may have contributed to an ICI-sensitive phenotype despite low PD-L1 expression.

These observations emphasize the need to expand predictive biomarkers beyond PD-L1. Future research should incorporate multiomic profiling, including TMB, microsatellite instability (MSI), and immune tumor microenvironment analysis, to develop more accurate immunotherapy selection criteria for BC patients. In addition to PD-L1 expression, emerging biomarkers such as radiomic features, circulating tumor DNA (ctDNA), and exosome-based markers hold promise in predicting immunotherapy responses in BC.

Radiomics involves extracting high-throughput quantitative features from medical imaging to assess tumor characteristics non-invasively. Studies have shown that radiomic features can predict TMB, an emerging prognostic biomarker for immunotherapy in BC [53]. For instance, specific radiomic signatures from imaging modalities like CT and MRI have been associated with TMB status, potentially guiding immunotherapy decisions [54].

ctDNA comprises tumor-derived fragmented DNA circulating in the bloodstream. Its analysis offers a minimally invasive approach to monitor tumor dynamics and detect genetic alterations associated with resistance to immunotherapy [55]. Elevated ctDNA levels have been correlated with disease progression, and specific mutations identified in ctDNA can inform about potential resistance mechanisms, thereby aiding in tailoring immunotherapy strategies [56].

Exosomes are extracellular vesicles that facilitate intercellular communication by transporting proteins, lipids, and nucleic acids. Tumor-derived exosomes can modulate the immune response and have been implicated in immune evasion mechanisms [57]. Analyzing the molecular cargo of exosomes, such as PD-L1 expression levels, may provide insights into the tumor microenvironment and help predict responses to immune checkpoint inhibitors [58].

Integrating these biomarkers with PD-L1 expression could enhance the predictive accuracy of immunotherapy responses in BC, leading to more personalized and effective treatment strategies [56].

Conversely, the recurrence of BC despite initial complete necrosis under Nivolumab may suggest the emergence of some occult resistance mechanism to immunotherapy. Given that PD-L1 expression further declined in the recurrent tumor (CPS = 1), the loss of antigen presentation may have been due to downregulated MHC class I expression, reducing tumor cell visibility to cytotoxic T cells, despite continued immunotherapy exposure [59]. In fact, tumors have been known to escape immune surveillance by activating compensatory inhibitory pathways, such as T-cell immunoglobulin and mucin-domain containing-3 (TIM-3), lymphocyte-activation gene (LAG)-3, or T-cell immunoreceptor with Ig and ITIM domains (TIGIT), which were not assessed in this study, but could be explored in future research on the upregulation of alternative immune checkpoints [25,60]. Lastly, changes in tumor-associated macrophage polarization may be responsible, with immunosuppressive M2 macrophages being able to counteract the effects of ICIs by fostering a tumor-friendly microenvironment [61,62].

Overall, this study provides several important clinical and translational insights:

- Vigilance for secondary malignancies in BRAF/MEK-treated patients: Clinicians should be aware of the potential for BC development during anti-BRAF/MEK therapy and ensure appropriate urological monitoring [63].
- PD-L1 alone is an imperfect biomarker for immunotherapy selection: The observed tumor regression despite low PD-L1 levels suggests that additional biomarkers (TMB, MSI, immune cell infiltration) should be considered in future clinical trials [14,60].
- Resistance to ICIs may evolve over time: The progressive decrease in PD-L1 expression in our case highlights the dynamic nature of tumor immune interactions, emphasizing the need for serial biomarker assessment in ICI-treated patients [55].

In the end, PD-L1 expression, quantified using the CPS, is widely used to guide immunotherapy decisions in BC. However, response to ICIs remains heterogeneous, and PD-L1 positivity alone does not always predict therapeutic benefit. In this case, the prenecrosis tumor exhibited a CPS = 5, while the postnecrosis recurrent tumor showed a further decline to CPS = 1, suggesting yet another paradox where tumor regression occurred despite lower PD-L1 expression levels.

To place these findings into a broader clinical context, existing literature indicates that 15–40% of advanced bladder cancers are PD-L1 positive (CPS ≥ 10), depending on the scoring system and cutoff used [64,65]. However, a substantial proportion of PD-L1-negative patients still respond to ICIs, suggesting that additional factors beyond PD-L1 status contribute to treatment efficacy, which may better predict response to immunotherapy than PD-L1 alone [60]. Moreover, given that this study is based on a single patient, the ability to draw definitive conclusions is inherently limited by a lack of statistical power. PD-L1 expression is known to be heterogeneous within tumors, and CPS scoring is influenced by tumor microenvironment dynamics. Furthermore, serial assessments of PD-L1 expression in larger cohorts have shown that expression levels can fluctuate over time, particularly in response to prior therapies such as anti-BRAF/MEK inhibitors, which could alter the tumor’s immunogenicity [59].

To validate the implications of low PD-L1 CPS in predicting response to Nivolumab, larger cohort studies should examine PD-L1 expression longitudinally in BC patients receiving ICIs to assess variability and analyze how prior therapies (e.g., targeted inhibitors) affect PD-L1 regulation and ICI sensitivity. In the meantime, other biomarkers, such as TMB, ctDNA, and immune cell profiling, must be integrated to refine response predictions. While this case highlights an unusual immunotherapy response despite low PD-L1 expression, further research is needed to establish whether specific BC subtypes exhibit similar paradoxical responses, thereby improving biomarker-driven treatment selection in clinical practice.

## 5. Conclusions

This study highlights the complex interplay between targeted therapy and BC oncogenesis. We report a unique clinical case where anti-BRAF/MEK therapy for *BRAF-V600E*-positive MM appeared to coincide with the emergence and progression of urothelial carcinoma, suggesting potential implications for these agents in bladder tumorigenesis. Simultaneously, the paradoxical response to Nivolumab, despite low PD-L1 expression, challenges current predictive models for immunotherapy. Conversely, the recurrence of BC despite initial complete necrosis under Nivolumab may suggest the emergence of some occult resistance mechanism to immunotherapy. These findings emphasize the need for more reliable biomarkers to guide immunotherapy decisions in BC. To date, no reliable predictive biomarkers have been clearly identified for immunotherapy response in urothelial carcinoma. Further research should focus on understanding alternative immune response mechanisms and the role of targeted therapy in BC evolution.

Lastly, our paper underlines the importance of interdisciplinary approaches and personalized treatment strategies in managing concurrent malignancies.

## Figures and Tables

**Table 1:** Summary of bladder cancer evolution under concomitant oncological/immunological treatments for pre-existing malignant melanoma.

Date	Procedure	Findings	Concomitant Treatment ^1^
12 July 2019	TURBT	- pTaG2 high grade; - muscularis propria absent.	Dabrafenib + Trametinib
2 June 2020	TURBT	- pTaG2 high grade, but with residual tumor; - muscularis propria present.	Nivolumab
9 July 2020	TURBT	- pT1G2 high grade (biopsy), i.e., large residual tumor.	Nivolumab
30 September 2020	Cistoscopy	- complete spontaneous tumor necrosis.	Nivolumab
1 February 2021	TURBT	- pT1G2 high grade, with focal squamous features; - muscularis propria absent.	Nivolumab
23 March 2021	Radical Cystectomy	- pTaG2 high grade, “early urothelial carcinoma”, R0; - N0 (34 lymph nodes); - incidental prostatic adenocarcinoma, GS 3 + 3 = 6, pT2a	Nivolumab

^1^ Concomitant oncological/immunological treatment for malignant melanoma; TURBT—transurethral resection of bladder tumor; p—pathological; GS—Gleason Score. The pre- and postspontaneous bladder tumor necrosis tissue fragments used for further investigations are marked in red.

## Data Availability

Data available on request.
